# Human-to-Computer Interactivity Features Incorporated Into Behavioral Health mHealth Apps: Systematic Search

**DOI:** 10.2196/44926

**Published:** 2023-06-30

**Authors:** Ann Futterman Collier, Shelby Hagemann, Susan Brown Trinidad, Morgan Vigil-Hayes

**Affiliations:** 1 Research Department Southcentral Foundation Anchorage, AK United States; 2 School of Informatics, Computing, and Cyber Systems Northern Arizona University Flagstaff, AZ United States; 3 Department of Bioethics and Humanities University of Washington School of Medicine Seattle, WA United States

**Keywords:** app, behavioral app, behavioral health, consumers, engagement, health application, interactivity, mHealth, stickiness, support, therapeutic, user engagement, users

## Abstract

**Background:**

While there are thousands of behavioral health apps available to consumers, users often quickly discontinue their use, which limits their therapeutic value. By varying the types and number of ways that users can interact with behavioral health mobile health apps, developers may be able to support greater therapeutic engagement and increase app stickiness.

**Objective:**

The main objective of this analysis was to systematically characterize the types of user interactions that are available in behavioral health apps and then examine if greater interactivity was associated with greater user satisfaction, as measured by app metrics.

**Methods:**

Using a modified PRISMA (Preferred Reporting Items for Systematic Reviews and Meta-Analysis) methodology, we searched several different app clearinghouse websites and identified 76 behavioral health apps that included some type of interactivity. We then filtered the results to ensure we were examining behavioral health apps and further refined our search to include apps that identified one or more of the following terms: peer or therapist forum, discussion, feedback, professional, licensed, buddy, friend, artificial intelligence, chatbot, counselor, therapist, provider, mentor, bot, coach, message, comment, chat room, community, games, care team, connect, share, and support in the app descriptions. In the final group of 34 apps, we examined the presence of 6 types of human-machine interactivities: human-to-human with peers, human-to-human with providers, human-to–artificial intelligence, human-to-algorithms, human-to-data, and novel interactive smartphone modalities. We also downloaded information on app user ratings and visibility, as well as reviewed other key app features.

**Results:**

We found that on average, the 34 apps reviewed included 2.53 (SD 1.05; range 1-5) features of interactivity. The most common types of interactivities were human-to-data (n=34, 100%), followed by human-to-algorithm (n=15, 44.2%). The least common type of interactivity was human–artificial intelligence (n=7, 20.5%). There were no significant associations between the total number of app interactivity features and user ratings or app visibility. We found that a full range of therapeutic interactivity features were not used in behavioral health apps.

**Conclusions:**

Ideally, app developers would do well to include more interactivity features in behavioral health apps in order to fully use the capabilities of smartphone technologies and increase app stickiness. Theoretically, increased user engagement would occur by using multiple types of user interactivity, thereby maximizing the benefits that a person would receive when using a mobile health app.

## Introduction

### Overview

During the COVID-19 pandemic, policy makers, health care providers, and health advocates observed staggering gaps in access to behavioral health care that negatively impacted people around the globe [1]. The term “behavioral health” encompasses mental health and substance abuse, life stressors and crises, and stress-related physical disorders. It has also been used to refer to eating disorders, gambling, and sex addictions. Behavioral health apps are a form of mobile health (mHealth) technology that can help users independently manage their emotional well-being. Such apps represent an intriguing mechanism to bridge gaps in access to behavioral health care and education [1]. Several systematic reviews support the use of behavioral health apps in both youth and adults, especially for the treatment of anxiety, depression, substance abuse, and eating disorders [2-9]. There are now an estimated 10,000-20,000 behavioral health apps available to consumers [2].

Behavioral health apps vary in what they offer, ranging from self-help resources to web-based care to therapy augmentation [10] and they can span different stages of clinical care. Temkin and colleagues [5] categorized behavioral health apps as falling into 1 of 2 categories: those that emphasize assessment and those that emphasize treatment. Assessment apps focus on data that can be used to monitor symptoms, thoughts, moods, and behaviors and are especially useful in clinical settings. Assessment is often the first step in evidence-based treatment and offers insights that can have meaningful therapeutic and self-monitoring value. Treatment apps, alternately, focus more specifically on building behavioral health skills and reinforce therapeutic techniques; they typically include teaching the user mindfulness, meditation, and cognitive and behavioral techniques [6,9,11].

Despite the effectiveness of behavioral health apps, data suggest that once a behavioral health app has been downloaded, most users rapidly discontinue its use [5]. This likely prevents the app from achieving its full therapeutic value [12-15]. Reasons for discontinuation are largely unknown, but it has been speculated that user attenuation is associated with poor usability, boredom, and data-entry burden [5,14,16]. In addition, studies suggest that many behavioral health apps are not designed with service users in mind, do not solve problems users care most about, do not respect privacy, are not seen as trustworthy, and are unhelpful during behavioral health emergencies [15]. Information technology investigators have used the term “stickiness” to describe a mobile app’s ability to hold user attention [17], keep consumers returning for regular use, and encourage app stickiness. Carlo and colleagues [11] operationalized stickiness as a quotient derived from the number of monthly active users per normalized total downloads, where a higher number indicates greater stickiness. Interestingly, Carlo et al [11] reported that out of 46 behavioral health apps, stickiness was not associated with total downloads; in fact, lesser-known apps were more often identified as the stickiest. Thus, popularity measured by total downloads may not be indicative of app stickiness.

Alternatively, stickiness in mental health apps may be more strongly associated with user engagement [18,19]. In the context of psychotherapy, greater engagement in therapy is associated with better outcomes after counseling interventions [20,21]. In the field of human-computer interactions, app engagement is often described as a function of how much a user invests cognitively, emotionally, and behaviorally in an app [22], with a heavy emphasis on the frequency and duration of use as well as popularity and user loyalty [23,24]. In general, app usage metrics are often heavily weighted in assessing involvement with the app through behaviors such as clicks, downloads, and time spent using an app, which may capture components of what can be defined as user engagement [24-26].

App engagement could also be examined through the way a user interacts with the app or website. While human-to-machine interactivity has been examined in behavioral health apps, it has been limited in scope and is typically described as the look and feel of the device or the software instead of explicit ways that people interacted with the app itself [12]. By varying the types and number of ways that users can interact with a behavioral health program, app developers may be able to support greater therapeutic engagement [24]. In this way, app interactivity would involve a wider array of relational dynamics.

In Table 1, we summarize the ways interactivity can be used in behavioral health apps. First, interactivity can provide assessment, captured as human-to-data interactions. Users are asked to record information such as mood ratings or alcohol use; the app then summarizes the data graphically. For example, a user would enter their daily alcohol intake, and the app would then produce a graph demonstrating use patterns over time. Interactivity can also include human-to-human interactions, where interactions occur between humans and are mediated by computer systems [16,27-29]. In human-to-human interactions, people can interact with providers, therapists, communities, and peer subgroups, for example, special interest group communities or direct messaging within the app [16]. Apps can also provide human-to–artificial intelligence (AI) interactions, which are defined as when a user interacts with an AI therapist or chatbot that provides therapeutic support. Human-to-algorithm interactions occur when the user interacts with software logic and protocols. For example, gaming interactivity uses multiple sensorimotor, motivational, and persuasion elements and supports cognitive processes. Human-to-algorithm interactions have been found to increase user enjoyment and motivation [30]. Human-to-algorithm interactions are not limited to gaming. They can include personalization features where the app receives input from a user through a questionnaire and then customizes the information, adapting the look and feel to match a user’s preferences. Behavioral health apps can also leverage the unique interactivity components that are specific to mobile devices, or what we refer to as novel interactive smartphone modalities. This can involve haptics, gestures and movement, and location such as through GPS, scan-and-tilt, point of view and head tracking, multitouch and video projection, context and proximity sensing, auditory input, and even augmented reality [31-36].

**Table 1:** Types of interactivities that can be used in behavioral health apps.

Type of interactivity	Definition of interactivity
Human-to-data	Provides and retrieves self-recorded data about behavior and symptoms, such as daily alcohol usage.
Human-to-human (peers or provider)	Human interactions mediated by computer systems (eg, community boards and direct messaging) with peers (or community of users) or with providers, defined as a coach, paraprofessional, or professional.
Human-to–artificial intelligence	Users interact with an artificial intelligence, such as a chatbot, to provide therapeutic support.
Human-to-algorithm	Users interact with software logic and protocols, allowing for personalization of the app or gaming. Can use multiple sensorimotor, motivation, and persuasion elements and supports cognitive processes.
Interactive smartphone modalities	Involves haptics, gestures and movement, locations, scan-and-tilt, point of view and head tracking, multitouch and video projection, context and proximity sensing and auditory input, and even augmented reality.

We were unaware of any behavioral health apps that included all the interactivity features described in Table 1. This may be because designers use technology to deliver information in more traditional formats rather than offering creative, novel, and engaging mental health interactions [8]. For example, app developers could use mHealth tools to deliver content in a web-based forum through lesson manuals instead of revamping the interventions to take advantage of the unique capabilities that an app can offer. As such, behavioral health apps may be more effective if the delivery approach uses the full range of potential app interactivities. This assumes that greater interactivity would then be associated with increased user engagement, allowing the app to have a greater impact. For example, an app that involves therapist-to-user interactivity (human-to-human) would allow the provider to see what the user was doing in the app, possibly hold the user accountable for his or her behaviors, allow the therapist to recommend specific in-app activities for the user to try (human-to–physical device), as well as have the user review and record their mood and behaviors (human-to-data). An app that included gamification (human-to-algorithm) could further incentivize intended behaviors by rewarding the user and making the activities more enjoyable, as well as personalizing the activities presented for the user. We wondered if a greater variety of in-app interactivity would be associated with greater enjoyment of and engagement with an app, thereby promoting more regular involvement with the intended intervention. Although this hypothesis makes sense, evaluating the impact of interactivity on engagement would require empirical testing. To date, no studies have either experimentally or retrospectively examined app interactivity or summarized the types of interactivities that exist in behavioral health apps.

### Objective

The main objective of this analysis was to systematically characterize the types of user interactions that are available in behavioral health apps and then examine if greater interactivity was associated with greater user satisfaction, as measured by app metrics. By varying the types and number of ways that users can interact with behavioral health programs, app developers may be able to support greater therapeutic engagement and increase mHealth app stickiness [36]. To date, there are no guidelines for the conduct and reporting of systematic searches of mHealth apps [37]. As such, we adapted data elements from the PRISMA (Preferred Reporting Items for Systematic Reviews and Meta-Analyses) guidelines for systematic literature reviews [37,38]. We then surveyed existing behavioral health apps to determine the types and spectrum of interactivities offered to users. We expected to see a positive association between the number of interactivity “types” and app metrics indicating user satisfaction.

## Methods

### Search Strategy and Inclusion Criteria

**Figure 1:**
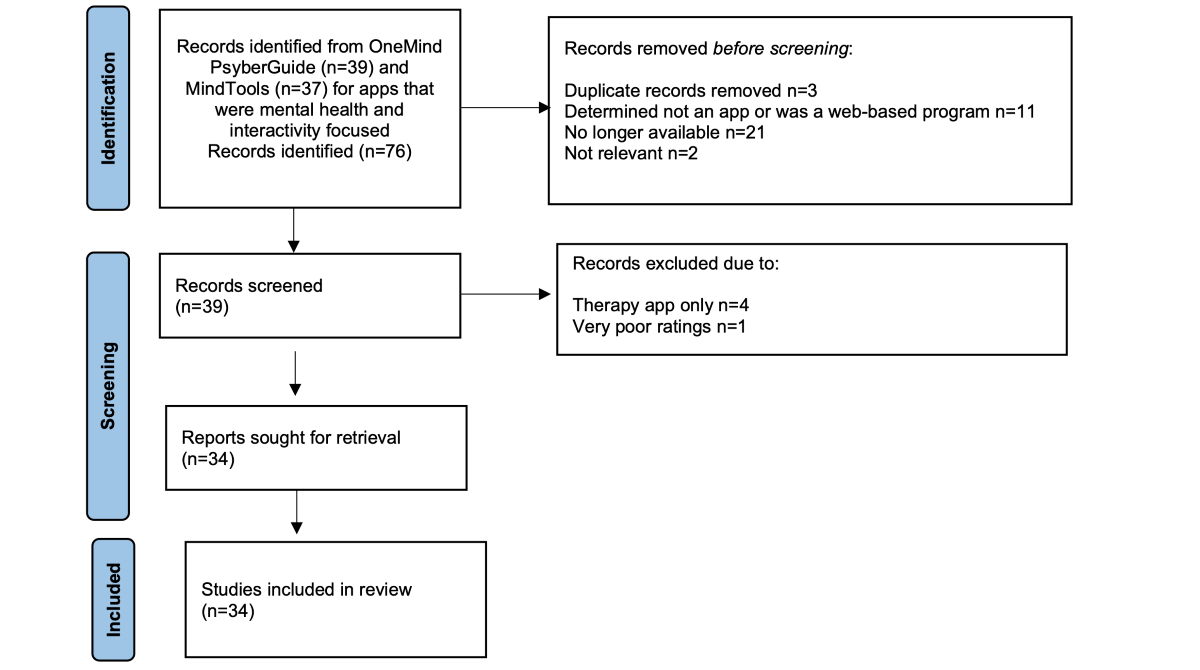
Identification of studies from mHealth clearinghouses.

App clearinghouse websites provide a means for professional review of the wide array of available behavioral health apps [39]. They allow for efficiency, whereby numerous apps can be summarized in 1 place, thereby providing access to systematic evaluations of app usability, functionality, and accuracy of content, and in some cases, continual updating as new apps become available [39]. In July 2021, we were aware of 5 mHealth clearinghouses for English-language behavioral health apps: ORCHA, Credible Minds, MindApps, Psyberguide, and MindTools. We developed tools to scrape data from all 5 of these clearinghouses but were unable to scrape from ORCHA, Credible Minds, and MindApps because they either required accounts to use them or blocked us from data scraping. Therefore, we scraped data from Psyberguide and MindTools (see Figure 1). We first filtered the results to ensure we were examining only behavioral health apps by looking for the phrases depression, stress, anxiety, panic attacks, relaxation, mood, mindfulness, fear, PTSD, and substance abuse. Our search was then refined to include apps that involved interactivity by including the following search terms: peer or therapist forum, discussion, feedback, professional, licensed, buddy, friend, AI, chatbot, counselor, therapist, provider, mentor, bot, coach, message, comment, chat room, community, games, care team, connect, share, and support in the app descriptions. We then merged the identified apps that met both our behavioral health and interactivity criteria across the MindTools (n=37) and Psyberguide (n=39) clearinghouses, resulting in a total of 76 unique apps.

After examining the 76 unique apps, 42 were removed because they met one or more of the following criteria: no longer available for download; insufficient information available either on the clearinghouse website or internet about the app; closer examination revealed that the app did not involve behavioral health concerns; the app was not actually an app but a web-based program; or the app was identified as having the sole purpose of directing a person to a therapist (eg, TalkSpace). In some cases, clearinghouse reviews about the app identified serious concerns, such as little information regarding the app’s functionality, accessibility, or security; these apps were eliminated from further consideration. We reviewed the remaining 34 apps.

### Coding of App Elements and Data Collection

#### Coding and Data Collection

Three apps were initially evaluated by 3 authors to develop consensus on how to conduct the ratings. The remaining apps were evaluated by author SH alone. Descriptive data were obtained and recorded in a SurveyMonkey questionnaire and included the following: app name, platform (Apple App, Google Play store, etc); average user rating from the Apple App store; affiliation (commercial, government, nongovernmental agency, university, unknown, etc); platform used (iPhone, iPad, Android, or other); country of origin; and whether the app was geared toward children or adults. We also used 3 items from the Mobile Application Rating Scale (MARS) [40] to evaluate (1) data privacy and security (Health Insurance Portability and Accountability Act [HIPAA], used in the United States, or Data Protection Act [DPA], used in England); (2) behavioral health goals of the app; and (3) psychological models and strategies purportedly used by each app.

#### Interactivity

We evaluated the presence or absence of the app interactivity types defined in Table 1. We counted human-to-human scores for peers and for providers separately to distinguish social versus professional support. A summary score for interactivity was created by summing all the types of interactivities possible, which ranged from 0 to 6.

#### User Ratings, Visibility Scores, and Total Downloads

User ratings for each of the apps were collected directly from the Apple App store. The estimated downloads and visibility scores were also collected from each app’s history before November 21, 2021, which was the day of data collection. User ratings are based on user feedback and reviews and indicate the overall quality and user satisfaction of an app. These scores range from 1 (lowest) to 5 (highest), with 3 representing general satisfaction. User visibility scores provide information about how easily an app can be found in an app store search. They range from 0 to 100; a visibility rating around 35%-45% is considered strong, as this generally brings the app to one of the top positions in the search results. Total downloads refer to the total number of users that have downloaded an app on a mobile device, usually a mobile phone, from an app store. The metric combines first-time downloads with app store redownloads.

### Statistical Analysis

A PRISMA 2020 flow diagram was used to map the different phases of the app review [38]. Statistical analyses were conducted using SPSS for Windows (version 27.0; IBM Corp). Descriptive statistics were used to summarize percentages, means, and SDs for each type of interactivity observed. Pearson correlation coefficients were calculated to determine the association between the total number of interactivities in an app and the user ratings, user visibility score, and total downloads.

### Ethical Considerations

As the study was determined not to involve human subject research, it did not undergo institutional review board review and involved no informed consent procedures, privacy or confidentiality protections, or participant compensation.

## Results

### General Characteristics

Most of the 34 (88%) apps reviewed were available for download on both the Apple App and Google Play (Android) stores; 4 apps (Cognifit, iMood Journal, MoodKit, and VetChange) were available only on the Android platform.

We determined that 53% (18/34) of the apps were developed in the United States. Only 1 was geared toward children younger than 12 years; most either indicated that they could be used with youth 13 years and older or did not specify an age range. For most of the apps (19/34, 55.9%), the rater could not determine the country of origin. It appeared that 11 (32.4%) apps were developed for commercial purposes only, and 2 (5.9%) were developed by a government organization. We identified that 14.7% (5/34) of the apps were HIPAA or DPA compliant; 26.5% (9/34) were not. In 58.8% (20/34) of the cases, we could not determine if the app was HIPAA or DPA compliant. The average rating for data security was 2.2 (SD 1.47), with scores ranging from 0 to 4.

Using the MARS categories, the most common behavioral health goals for the reviewed apps involved happiness (15/34, 44.1%), anxiety reduction (15/34, 44.1%), stress management (15/34, 44.1%), emotional awareness (14/34, 41.2%), mindfulness and self-awareness (13/34, 38.2%), goal setting (13/34, 38.2%), reduction of negative emotions (12/34, 35.3%), and reduction of depression (9/34, 26.5%). The psychological models and strategies used in the reviewed apps predominantly involved monitoring and tracking (22/34, 64.7%), informational conveyance (15/34, 44.1%), goal setting (14/34, 41.2%), advice giving (14/34, 41.2%), assessment (13/34, 38.2%), cognitive-behavioral therapy (9/34, 26.5%), relaxation (9/34, 26.5%), and mindfulness (8/34, 23.5%).

### App Interactivity

Please see Table 2 for a review of the interactivity features included in the 34 apps. The average number of interactivity features included in each app was 2.53 (SD 1.05; range 1-5). None of the apps used all 6 of the possible interactivity features. The 2 apps with the greatest number of interactivity features were Sanvello: Anxiety & Depression and Connections, both using 5 interactivity features. Four apps (7 Cups, The DayBreak app, Recovery Record, and SuperBetter) included 4 interactivity features.

**Table 2:** Interactivities observed in behavioral health apps (N=34).

	Human-human: peer	Human-human: provider	Human–artificial intelligence	Human-algorithm	Human-data	Smartphone features	Total interactive features per app^a^
365 Gratitude Journal, n	1	0	0	1	1	0	3
7 Cups, n	1	1	1	0	1	0	4
BoosterBuddy, n	0	0	1	1	1	0	3
BrainHQ, n	0	0	0	1	1	1	3
Calm, n	0	0	0	0	1	1	2
CBT-i Coach, n	0	0	0	0	1	0	1
CogniFit, n	0	0	0	1	1	0	2
Connections/CHESS Health, n	1	1	0	1	1	1	5
The Daybreak app (Hello Sunday Morning), n	1	1	0	1	1	0	4
Fabulous: Motivate Me! Meditate, Relax, Sleep, n	1	0	0	0	1	0	2
Fit Brains Trainer, n	0	0	0	1	1	0	2
Habitica: Gamify Your Tasks, n	0	0	0	1	1	0	2
Headspace: Mindful Meditation, n	1	0	0	0	1	1	3
iMoodJournal-Mood Diary, n	0	0	0	0	1	0	1
Insight Timer – Meditation, n	0	0	0	0	1	1	2
Liberate: My OCD Fighter, n	0	1	0	0	1	0	2
Lumosity: Brain Training, n	0	0	0	1	1	0	2
MoodKit, n	0	0	0	0	1	0	1
Muse: EEG Meditation & Sleep, n	0	0	1	0	1	1	3
MyLife Meditation, n	0	0	0	1	1	0	2
My QuitBuddy, n	1	0	0	0	1	0	2
Peak – Brain Training, n	0	0	0	1	1	0	2
PTSD Coach, n	0	1	0	0	1	0	2
Recovery Record (RR: Eating Disorder Management; Nourishly-Nutrition & Diet), n	1	1	0	1	1	0	4
Rise Up + Recover, n	0	0	0	0	1	1	2
Sanvello: Anxiety & Depression, n	1	1	0	1	1	1	5
Serenita—Stress & Anxiety, n	0	0	0	1	1	1	3
Stay Quit Coach, n	0	0	0	1	1	0	2
Step Away: Alcohol Help, n	0	0	1	0	1	0	2
SuperBetter, n	1	1	1	0	1	0	4
VetChange, n	0	0	0	0	1	0	1
Virtual Hope Box, n	0	1	0	0	1	1	3
Woebot: Your Self-Care Expert, n	0	0	1	0	1	0	2
Wysa: Mental Health Support, n	0	1	1	0	1	0	3
Total interactive features observed, n (%)	10 (29.4)	10 (29.4)	7 (20.5)	15 (44.2)	34 (100)	10 (29.4)	N/A^b^

^a^The mean number of interactive features per app was 2.53 (SD 1.05).

^b^N/A: not applicable.

All of the apps allowed for human-to-data interactions. After human-to-data interactions, human-to-algorithm features were the most frequently included type of interaction (15/34, 44.2%). When gaming was used, users were encouraged to participate in goal tracking and practice challenges (eg, daily or monthly), for which they received some type of reward (points, stamps, battle monsters, swords, or medals) for their participation. Gaming features also involved congratulations and positive reinforcement for goal accomplishment, as well as having content tailored to the user based on their expressed interests in screening quizzes (Fabulous Motivate Me!, Stay Quit Coach, 365 Gratitude Journal, and Sanvello: Anxiety & Depression). None of the apps reviewed included interactive gaming features with other users or AI; gaming was strictly an interaction between the user and the app.

The next most popular interactivity feature used was human-to-human (provider) interactivity (10/34, 29.4%), where the user could interact with a professional or paraprofessional. Following this category was human-to-human (peers) interactivity (10/34, 29.4%), where the user could interact with peers through community boards or in-app messaging. Only 6 (17.6%) of the apps allowed access to both professionals and peers. The least frequently used type of interactivity was human-to-AI, where 7 (20.5%) of the apps allowed the user to interact with some form of AI, such as a chatbot.

Although 29.4% (10/34) of the apps used components unique to smartphone technology, most apps (8/10, 80%) using this feature involved the ability to listen to audio content, specifically music, meditations, and podcasts. Two apps, Serenita—Stress & Anxiety and Muse: EEG Meditation & Sleep, incorporated a form of biofeedback that relied on smartphone technology. Where Serenita—Stress & Anxiety included a “stress check” that measured stress levels through the user’s phone camera, Muse: EEG Meditation & Sleep offered a system for users to measure electric brain rhythms during meditation with a connected headband. None of these apps used haptics, augmented reality, or geolocation.

### Features of the Top-Rated Apps

Apple app user ratings (Table 3) for these 34 apps ranged from 2.0 to 5.0, with the average rating at 4.49 (SD 0.29). In Table 3, the top-ranking apps are noted, with ties for first, second, and third place. The apps rated in first place (with 5.0 user ratings) were Insight Timer Meditation, Recovery Road, VetChange, and WYSA. In second place (with ratings of 4.9) were 7 Cups, Calm, Headspace: Mindful Meditation, MyLife Meditation, Sanvello: Anxiety & Depression, and Woebot: Your Self-Care Expert. In third place (with 4.8) were 365 Gratitude Journal, BoosterBuddy, Lumosity: Brain Training, Peak—Brain Training, Rise Up + Recover, and SuperBetter. The 2 lowest-rated apps were Stay Quit Coach Legacy and Serenita—Stress & Depression.

The number of downloads ranged from 8 (Cognifit) to 667,000 (Calm); the average number of downloads was 58,600 (SD 17,0776.9). Because many of the apps did not have download information available, the score was not used for data analysis. The average visibility score was 59.7 (SD 15.77; range 27-89). Interactivity was not significantly correlated with Apple user ratings (*r*=0.149; *P*=.40) nor with the visibility score (*r*=–0.004; *P*=.98). The visibility score was significantly correlated with user ratings (*r*=0.598; *P*<.001).

**Table 3:** Apple store user ratings, visibility scores, and total downloads for behavioral health apps reviewed (N=34).

Name of app	User rating	Visibility score	Total downloads, n
365 Gratitude Journal	4.8 (3)^a^	65	367
7 Cups	4.9 (2)^a^	67	1300
BoosterBuddy	4.8 (3)^a^	40	47
BrainHQ	4.5	62	123
Calm	4.9 (2)^a^	89	667,000
CBT-i Coach	3.5	63	8
Cognifit	4.5	60	514
Connections/CHESS Health	4.1	46	—^b^
The Daybreak app (Hello Sunday Morning)	4.1	43	—
Fabulous: Motivate Me! Meditate, Relax, Sleep	4.6	84	47,000
Fit Brain Trainer	4.0	50	—
Habitica: Gamify Your Tasks	4.0	82	812
Headspace: Mindful Meditation	4.9 (2)^a^	86	548,000
iMoodJournal-Mood Diary	4.1	46	3000
Insight Timer - Meditation	5.0 (1)^a^	79	62,000
Liberate: My OCD Fighter	4.5	64	—
Lumosity: Brain Training	4.8 (3)^a^	81	40,000
MoodKit	4.1	50	1700
Muse: EEG Meditation & Sleep	4.5	59	578
MyLife Meditation	4.9 (2)^a^	72	2500
My QuitBuddy	4.5	47	614
Peak – Brain Training	4.8 (2)^a^	74	15,000
PTSD Coach	4.6	61	164
Recovery Record (RR: Eating Disorder Management; Nourishly-Nutrition & Diet)	5.0 (1)^a^	64	1200
Rise Up + Recover	4.8 (3)^a^	63	—
Sanvello: Anxiety & Depression	4.9 (2)^a^	71	10,000
Serenita- Stress & Anxiety	3.1	27	—
Stay Quit Coach	2.9	34	—
Step Away: Alcohol Help	4.7	41	—
SuperBetter	4.8 (3)^a^	53	—
VetChange	5.0 (1)^a^	36	—
Virtual Hope Box	4.0	46	167
Woebot: Your Self-Care Expert	4.9 (2)^a^	69	2300
Wysa: Mental Health Support	5.0 (1)^a^	75	2000

^a^The top 3 apps are ranked with (1), (2), and (3). When a numerical rating is listed more than once, this was because of ties.

^b^Not available.

## Discussion

### Overview

While there are purportedly more than 10,000 behavioral health apps available for consumers to download [2], our results suggest that most apps fail to leverage the unique capabilities of the app platform; none of the 34 behavioral health apps we reviewed used the full range of interactivity features afforded by smartphone and tablet technology. On average, the apps reviewed included fewer than 3 interactivity features. The most common types of interactivities included were human-to-data, human-to-algorithm, human-to-human (professional), and human-to-human (peer). Assessment, referred to herein as human-to-data interactivity, has been previously identified as a commonly included feature in behavioral health apps [6]. The least common type of interactivity used was human-to-AI. Very few apps used any of the novel interactive smartphone modalities available, including gestures and movement, locations, scan-and-tilt, point of view and head tracking, multitouch and video projection, context and proximity sensing, auditory input, and haptics. None of the apps reviewed supported human-to-algorithm (ie, gaming) activity with other users, and the apps rarely used both types of human-to-human interactivity, that is, enabling users to interact with both peers and providers.

Apps with the greatest number of interactivity features were Sanvello: Anxiety & Depression, Connections, 7 Cups, The DayBreak app, Recovery Record, and SuperBetter. Although these 5 apps had strong user ratings, Recovery Record was both ranked as one of the highest-rated apps and also had multiple interactivity features. There were no significant associations between the number of interactivity features and user ratings of the app or app visibility, leading to a lack of clarity about whether users appreciated these features.

Most apps we reviewed were available for download on both the Apple and Google Play stores, and the majority were developed for adults. Even though we looked for English-language apps, the country of origin where the app was designed was typically unspecified. Unfortunately, more than half of the apps did not state if they were HIPAA or DPA compliant, and security ratings were quite low on average. App developers usually did not specify whether the app had been developed for commercial or nongovernmental agencies.

To our knowledge, no previous study has examined these 6 types of interactivities in behavioral health apps. Studying 30-day user engagement with behavioral health mHealth apps, Baumel and Kane [12] did measure user engagement with attention to how content was presented, the types of interactive features that required user input and reaction, user irritation, the extent to which the intervention was targeted to a particular user context and personalized, and the extent to which the intervention piqued users’ interest and curiosity [12]. While Baumel and colleagues’ [13] definitions of engagement were operationalized based on evidence-based strategies, their scale lacked rigorous definitions, especially regarding how interactivity was specifically manifested in behavioral health mHealth apps. In addition, their definition of human and machine interactivity did not describe the full range of ways that humans can interact with an app.

The strength of our findings was expectedly dampened due to the small number of apps we found that truly included interactivity. The apps we reviewed predominantly targeted increasing positive mood, anxiety and stress management, emotional awareness, and mindfulness, and reducing negative emotions and depression. The theoretical approaches used in the apps evaluated were typically symptom monitoring and tracking, information conveyance, goal tracking, advice giving, and assessment. This is consistent with what others have reported in the literature [5]. The mHealth apps we reviewed predominantly relied on a behavioral approach toward reducing negative mood and stress and increasing positive mood. They frequently used an “expert” educational approach (ie, the app) by teaching the user through self-monitoring of symptoms. Research does suggest that apps with the strongest behavioral focus appear to have the greatest adherence [10]. When gaming was used, it was predominantly used as a means to reinforce symptom tracking or support small behavioral changes. While mindfulness and cognitive behavioral approaches are popular in psychology and with the lay public [6,9,11], they were not fully used in the mHealth apps we reviewed and certainly not integrated with the full range of interactivities available on mobile technology. For example, in addition to “teaching” a user the nuts and bolts of how to use cognitive-behavioral approaches for emotion management, a chatbot could have offered the user practice in breaking down activating events, behaviors, and consequences, and then community board messaging with human-to-human (peers) and human-to-human (provider) interactions could have further supported the application of theory. Instead, opportunities for active experiential learning were limited in range.

### Limitations

We had originally intended to examine the relationship between interactivity features and user engagement as defined through downloads and monthly and average daily use. We quickly realized that these data were not readily available because of changes in data privacy and transparency rules. As we engaged in our critical review of behavioral health apps, we found that commercial app stores (Apple Play Store and Google Play) hide critical data from users and health professionals. Information presented through app stores is dynamic and lacks information about the evidence that drives intervention features in the app, making it difficult to ascertain whether an app is appropriate and useful for the user. Moreover, these commercial repositories prevent researchers from accessing data that is necessary for formal assessment, including information about the number of downloads, churn, and retention. As such, our review was limited to the description of behavioral health app interactivity types, user ratings, visibility scores, and total downloads. Because we could not examine the relationship between interactivity features and daily or monthly average use, we were unable to create a stickiness quotient or app adherence as done by Carlo et al [11].

Another limitation of this study is that our assumption that greater interactivity will be associated with user engagement could be flawed. There may be a cognitive burden that occurs when operating a complex app, which could lead to less interest and engagement over time, and hence lower long-term use. Given that behavioral health app users may have mental health disorders, including depression, this is an important consideration.

### Conclusions

We put forth a novel idea that app developers would do well to include more interactivity features in apps and fully use the capabilities of smartphone technologies to increase app engagement. Theoretically, increased engagement would occur through the multiple types of user interactivity, thereby maximizing the benefits that a person could receive when using an mHealth app. Research is needed to allow investigators to directly examine the benefits of including multiple interactivity features, as they are more costly to produce. For example, experimental studies could examine mental health and well-being outcomes in people who used multiple interactivity features in behavioral health apps. Alternatively, users could provide actual ratings of the interactivity features they tried and then rate their engagement for a given day. Then, investigators could study ratings in conjunction with daily and weekly app use patterns. This would allow investigators to better understand the usefulness of these types of activities and the impact on mental health and to understand app therapeutic engagement at a more granular level. This study is one of the first to examine interactivity in behavioral health apps and found that most mHealth apps underuse interactivity features.

## Abbreviations

AI: artificial intelligence
DPA: Data Protection Act
HIPAA: Health Insurance Portability and Accountability Act
MARS: Mobile Application Rating Scale
mHealth: mobile health
PRISMA: Preferred Reporting Items for Systematic Reviews and Meta-Analyses
